# Identification and cytochemical immunolocalization of acetyl-CoA acetyltransferase involved in the terpenoid mevalonate pathway in *Euphorbia helioscopia* laticifers

**DOI:** 10.1186/s40529-017-0217-3

**Published:** 2017-12-16

**Authors:** Meng Wang, Dou Wang, Qing Zhang, Jia Chai, Yong Peng, Xia Cai

**Affiliations:** Key Laboratory of Resource Biology and Biotechnology in Western China, (Northwest University), Ministry of Education, Xi’an, 710069 China

**Keywords:** Terpenoid, Acetyl-CoA acetyltransferase, Gene clone, Prokaryotic expression, Expression, Immunolocalization

## Abstract

**Background:**

Terpenoids, the largest class of natural products in the plant kingdom, have been widely used in medicine. The precursors of terpenoids, isoprene phosphate (IPP) and dimethylallyl pyrophosphate (DMAPP), were synthesized from a mevalonate (MVA) pathway and a 2-C-methyl-d-erythritol-4-phosphate (MEP) pathway respectively. The acetyl-CoA acetyltransferase (AACT) is the initial enzyme in MVA pathway and is considered presently to be essential for terpenoid backbone biosynthesis. The basic research on cytochemistry of terpenoid metabolic enzymes is important for understanding the mechanisms underlying major metabolic processes. However, compartmentalization of AACT in plants is in controversy. *Euphorbia helioscopia* L. containing laticifers in the whole plant is a famous ancient folk medicine for tumor treatment, and the terpenoid is an active ingredient. Furthermore, the laticifer cell is the main synthesizing and storing site for terpenoids.

**Results:**

The gene of *AACT* was cloned successfully from *E. helioscopia*, and named as *EhAACT*. The *EhAACT* expression has no significant difference among roots, stems and leaves. However, compared with the roots and stems, the *EhAACT* expression level is slightly higher in leaves. In addition, EhAACT recombinant protein was expressed by procaryotic expression system and anti-EhAACT antibody was prepared, the molecular weight is about 43 kDa. Western blotting results illustrated that the EhAACT antibodies specifically recognized the endogenous proteins in *E. helioscopia* laticifers. At last, the subcellular localization of EhAACT in *E. helioscopia* laticifers was observed by using colloidal gold immune-electron microscopy. EhAACT was found to mainly distribute in the endoplasmic reticulum (ER), vacuoles originated from ER and cytosol aound vacuoles originated from ER.

**Conclusions:**

As a result, we speculated that in *E. helioscopia* laticifers, EhAACT located in cytosol would be transferred to small vacuoles dilated from ER, and the precursors of terpenoids were synthesized in these small vacuoles, then terpenoids were further synthesized into latex particles. This result would provide theoretical basis for regulating and controlling of terpenoid biosynthesis in laticifers.

## Introduction

Terpenoids are the largest class of natural products in the plant kingdom. Their structural unit is isoprene, and the general formula is (C_5_H_8_)_n_. Terpenoids have potential medicinal value and are widely used in the medicine, for example, paclitaxel, β-elemene, beta ionone and geraniol are used as anticancer drugs (Eisenreich et al. 1998; Hua and Cai 2006; Duncan et al. 2004). The precursors of terpenoids are five carbon compounds which are isoprene phosphate (IPP) and dimethylallyl pyrophosphate (DMAPP), these two kinds of precursors were synthesized from a mevalonate (MVA) pathway (Chappell 1995) and a 2-C-methyl-d-erythritol-4-phosphate (MEP) pathway (Lange et al. 1998) respectively. Compartmentalization of MVA pathway enzymes has been extensively studied in mammalian (Kovacs et al. 2002; Hogenboom et al. 2004; Wanders and Waterham 2006). In plants, there only exists fragmented experimental data regarding the localization of the MVA pathway enzymes (Sapir-Mir et al. 2008). Rodríguez-Concepción and Boronat (2015) pointed out that future efforts to address the regulation of isoprenoid metabolism must also take into account the spatial distribution of the biosynthetic pathways as well as the targeting and subcellular location of the enzyme isoforms involved, the intracellular transport of metabolic intermediates, and the different storage structures for end products (Rodríguez-Concepción and Boronat 2015). The acetyl-CoA acetyltransferase (AACT), or named acetoacetyl-CoA thiolase (ACAT) is a kind of enzyme that catalyzes hydrolysis and condensation. In the MVA pathway, AACT is the initial enzyme to catalyze two acetyl CoA to produce acetyl acetyl CoA and is essential for terpenoid backbone biosynthesis. Soto et al. (2011) found that acetoacetyl-CoA thiolase is a regulatory enzyme in isoprenoid biosynthesis involved in abiotic stress adaptation (Soto et al. 2011). Jin et al. (2012) considered that the AACT2-derived acetoacetyl-CoA pool generates isoprenoids which are required for normal growth and development, and these cannot be generated from other compensatory isoprenoid biosynthetic pathways (Jin et al. 2012). Therefore, AACT is highly significant for basic researches on the cytochemistry of terpenoid synthesis and the mechanisms underlying major metabolic processes. Furthermore, the localization of AACT in plants has been also in controversy.

*Euphorbia helioscopia* L. is a biennial herb of genus *Euphorbia* (Euphorbiaceae). The whole plant contains laticifers from which the white latex flowed out when the plant was broken. As an ancient folk medicine for tumor treatment, its active ingredients are terpenoids (Cai et al. 1999; Wang et al. 2012). The laticifer cell is the main synthesizing and storing site for terpenoids in *E. helioscopia*. In the mature laticifers, latex particles of various sizes were accumulated in the latex (Cai et al. 2009).

As a result, in order to further understand the compartmentalization of AACT, synthesis site, transportation and accumulation of the terpenoids in laticifers, we prepared AACT antibodies and analyzed the immunolocalization of EhAACT on the levels of proteins, cells and organelles in laticifers by using techniques of western blotting and colloidal gold immunoelectron microscopy. The results above would provide theoretical foundations for biosynthesis and regulation of terpenoids.

## Materials and methods

### Plant material

The healthy 3-month-old seedlings of *Euphorbia helioscopia* collected from the field at the Botanical Garden of Northwest University in Shaanxi Province (Shaanxi, People’s Republic of China) were used for RNA extraction.

### Molecular cloning of full length cDNA of *EhAACT*

Total RNA was extracted from stems, leaves and roots of three seedlings by the General Plant Total RNA Extraction Kit (BioTeke, China) according to the manufacturer’s instructions. The quality and concentration of RNA were determined by agarose gel electrophoresis and spectrophotometric analysis (Eppendorf, Germany). For cloning the conserved fragment of *EhAACT*, the cDNA was synthesized with a PrimeScript™ 1st Strand cDNA Synthesis Kit (Takara, China) following the manufacturer’s instructions. The primers of P1-S and P1-A were designed according to the conserved nucleotide sequences of *AACT* genes shared by other species (Table 1). A fragment of *EhAACT* was amplified by PCR using the cDNA as templates under the following conditions: 94 °C for 3 min followed by 30 cycles of amplification (94 °C for 30 s, 55 °C for 30 s, and 72 °C for 1 min), and a final elongation at 72 °C for 10 min. The amplified product was purified (Tiangen, China), ligated into a pMD 19-T Vector (Takara, China) and cloned in *Escherichia coli* strain DH5α followed by sequencing. For 3′-RACE of *EhAACT*, the two round 3′-RACE was carried out with a 3′-Full RACE Core Set (Takara, China). According to the manufacturer’s suggestion, an aliquot of 1 μg of total RNA was reverse transcribed to get the 3′ RACE-Ready cDNA with 3′-RACE Adaptor (provided in the kit). The Outer 3′-RACE PCR was first performed with primer P2-S (Table 1) and 3′-RACE Outer Primer (provided in the kit) under the conditions of 94 °C for 3 min followed by 35 cycles of amplification (94 °C for 30 s, 56 °C for 30 s, and 72 °C for 2 min), and a final elongation at 72 °C for 10 min. Using the Outer PCR products as templates, the Inner 3′-RACE PCR was performed with primer P3-S (Table 1) and 3′-RACE Inner Primer (provided in the kit) at the same reaction condition described above. The amplified product was purified, ligated into a pMD 19-T Vector and cloned in *E. coli* strain DH5α followed by sequencing. For 5′-RACE of *EhAACT*, the 5′ RACE-Ready cDNA was synthesized by reverse transcripting 2 μg of total RNA with 5′-CDS primer A and SMART II Aoligo supplied in the SMARTer™ RACE cDNA Amplification Kit (Clontech, USA). The Outer 5′-RACE PCR was performed with primer P2-A (Table 1) and Universal Primer A Mix (UPM, provided in the kit) at 94 °C for 3 min, followed by 25 cycles of amplification (30 s at 94 °C, 30 s at 68 °C and 2 min at 72 °C), and then 10 min extension at 72 °C. The product was used as templates for Inner PCR, which was performed with primer P3-A (Table 1) and Nested Universal Primer A (NUP, supplied in the kit) under 20 cycles of amplification (30 s at 94 °C, 30 s at 68 °C and 2 min at 72 °C). The final product was ligated into pMD19-T vector and cloned in *E. coli* strain DH5α followed by sequencing.

**Table 1:** Sequences of PCR primers used in this study

Target code	Sequence (5′ → 3′)	Purpose
P1-S	AATGACTTTGGAATGGGAGTTTG	Conserved fragment cloning
P1-A	TAAATAGTTCAGGAGCCTGAGC	Conserved fragment cloning
P2-S	GGCTTAGGAAAGTTTGATGCTG	3′-RACE outer PCR
P3-S	CTGTTACTGCTGGAAATGCCTCTA	3′-RACE inner PCR
P2-A	CCGCATCACCATATCCACGTATTCTAG	5′-RACE outer PCR
P3-A	GTGCAGATATTCCACGCTCAAAGC	5′-RACE inner PCR
P4-S	ATTCCCCTTTCCTTCAATCTCAG	Full-length cDNA cloning
P4-A	TGTAACAGACAGAACAGGATGGC	Full-length cDNA cloning
Actin-S	GGTAACATTGTGCTCAGTGGTGG	Reference gene
Actin-A	AACGACCTTAATCTTCATGCTGC	Reference gene
P5-S	GCAGGACGAGGAAAATCATC	Quantitative real-time PCR
P5-A	CCAGCAGTAACAGAACCACC	Quantitative real-time PCR

At last, after comparing and aligning the sequences of 5′-RACE and 3′-RACE products, the coding sequence of *EhAACT* was obtained through RT-PCR with primers P4-S and P4-A (Table 1). 2 µL of 5′ RACE-Ready cDNA was used for the PCR in a total volume of 50 µL under the following conditions: 30 cycles of amplification (98 °C for 10 s, 55 °C for 5 s, 72 °C for 2 min). The final product was ligated into pMD19-T vector and cloned in *E. coli* strain DH5α followed by sequencing. Finally, the *EhAACT* sequence had been submitted to NCBI Genebank and the accession number is KP995935.

### Bioinformatics analysis

The cDNA sequence of *EhAACT* was compared online in the non-redundant peptide database at the National Center for Biotechnology Information (NCBI) (http://www.ncbi.nlm.nih.gov). A coding sequence was predicted by NCBI ORF Finder (http://www.ncbi.nlm.nih.gov/gorf/orfig.cgi) and compared with other *AACT* by NCBI BLAST (http://blast.ncbi.nlm.nih.gov/Blast.cgi). Subsequently, multiple alignment analysis was performed with DNAMAN 6.0 software. And a phylogenetic tree was constructed using MEGA 6.0 software by applying the neighbor-joining method and was corrected using Poisson correction method.

### Expression pattern analysis of *EhAACT* by real-time quantitative PCR

The expression level of *EhAACT* in roots, stems, and leaves were quantified with SYBR^®^ Premix Ex Taq™ kit (Tli RNaseH Plus) (Takara, Japan) in the CFX96™ Real-Time PCR System (Bio-Rad, United States). After an initial denaturation at 95 °C for 10 s, the PCR was carried out with 39 cycles of 95 °C for 10 s, 60 °C for 30 s, and 72 °C for 20 s. The 25 µL reaction mixture included 1 µL of cDNA templates, 12.5 µL of 2 × SBRY Premix ExTaq buffer, 9.5 µL of DEPC-treated water, and 0.4 µmol/L of P5-S and P5-A primers (Table 1). The specificity of PCR products were decided through the melting curve analysis. The relative expression levels were normalized according to the internal standard of *actin* gene using the 2^−ΔΔCt^ method as described by Livak and Schmittgen (Livak and Schmittgen 2001). Experiments were performed in triplicate, and the results were represented as mean values ± standard error (SE).

### Prokaryotic expression and antibody preparation

The plasmids were used for overexpressing 6 × His-AACT recombinant protein, which the full open reading frame of *EhAACT* was subcloned into, were transformed into Rosetta cells. The cells were grown at 37 °C for more than 16 h, and then induced by the addition of isopropyl-d-thiogalactopyranoside. Cells were collected by centrifugation and then ultrasonic broken in ice bath until solution becomes clear. The supernatant obtained after centrifugation was collected, filtered and performed affinity chromatography. Recombinant AACT proteins were purified as His fusion proteins using a nickel-nitrilotriacetic acid agarose column according to the manufacturer’s instructions. Different concentrations of imidazole (50, 100, 200, 300 and 500 mmol/L) were used to elute, and the best concentration of imidazole elution was determined by SDS-PAGE. Then the purified EhAACT recombinant protein was obtained.

Protein concentrations were determined with SDS-PAGE, and then the gel pieces containing the recombinant proteins were extracted and injected directly into health rabbits. Antibodies from the rabbits were affinity purified using Cyanogen bromide-activated sepharose (Sigma-Aldrich, USA) conjugated with recombinant proteins and the specificity of antibodies was proved by the ELISA and immunoblot (WB).

### Immunoblot analysis

The stems were cut using a scalpel, and white milky sap flowing from the holes of the stems was collected using plastic micropipettes. The sap was treated by TCA-Acetone precipitation methods, and the precipitation was then re-suspended in lysis buffer (7 mol/L urea, 2 mol/L thiourea, 4% CHAPS, 2 mmol/L EDTA, 2 mmol/L Tris, 1 mmol/L PMSF). Total proteins were separated by 12% SDS-PAGE and electrophoretically transferred onto nitrocellulose membrane. The nitrocellulose membrane was incubated with EhAACT antibodies (diluted 1/1000 with TBST) overnight at 4 °C. Following incubation with the secondary antibody (diluted 1/2000 with TBST), horseradish peroxidase conjugated immunodetection was detected using the enhanced chemiluminescence system (Tanon, Shanghai, China).

### Electron microscopy analysis

Stem buds were fixed in a solution of 0.25% glutaraldehyde and 4% paraformaldehyde in 0.1 mol/L phosphate buffer (pH 7.2) for 4 h at 4 °C. After rinsing, samples were dehydrated stepwise in an acetone series from 30 to 100% and embedded in LR White resin (Sigma-Aldrich, USA). ULtrathin sections (60–70 nm) collected on nickel grids (1-GN; 150 meshes) were washed with PBS for three times, and blocked in 3% BSA-TBST solution for 1 h at 37 °C. Subsequently, samples were incubated in anti-EhAACT antibodies at a dilution of 1:50 overnight at 4 °C, 37 °C for 1 h, and then washed with PBS three times. The primary antibody binding was detected using anti-rabbit IgG conjugated with Gold particles (10 nm; Bioss, Beijing, China). After washing with PBS and distilled water, the nickel nets were examined and photographed with JEM-1230 (TEM H-7650, Japan).

## Results and discussion

### Full-length cDNA sequence of *EhAACT* Gene

**Fig. 1:**
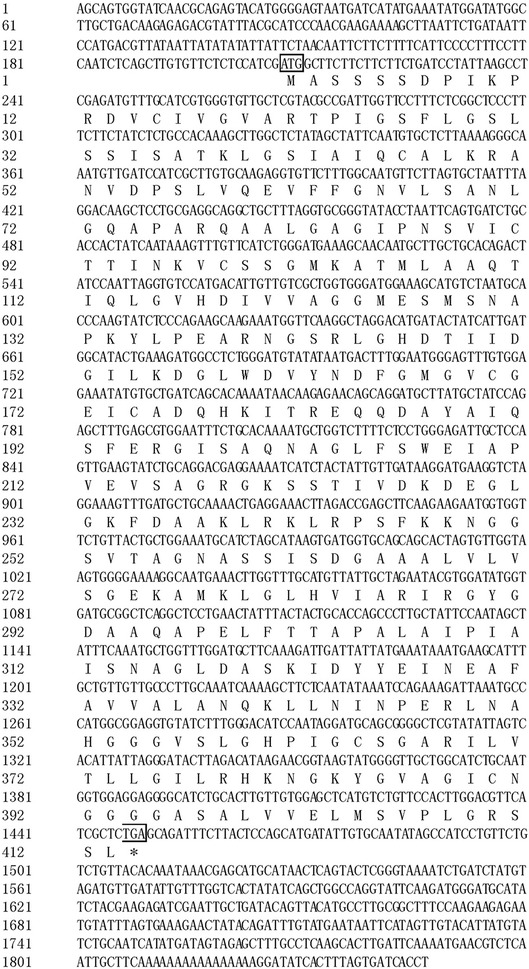
The full-length cDNA sequence of *EhAACT* and deduced amino acid sequence. “ATG” is initiation codon, “TGA” is termination codon

The full-length cDNA sequence of *EhAACT* was obtained by RT-PCR Using RACE method (GenBank Accession No. KP995935). It was found to be 1847 bp consisted of a 1239 bp coding sequence, a 207 bp 5′ untranslated region, and a 401 bp 3′ untranslated region (Fig. 1). It encoded a peptide of 413 amino acids. By using the software of Computer ProtParam Tool at http://web.expasy.org/protparam, the calculated molecular weight and isoelectric point (pI) of the deduced EhAACT protein were predicted to be 42.61 kDa and 8.7, respectively.

### Homologous analysis, multiple sequence alignment and phylogenetic tree construction

**Fig. 2:**
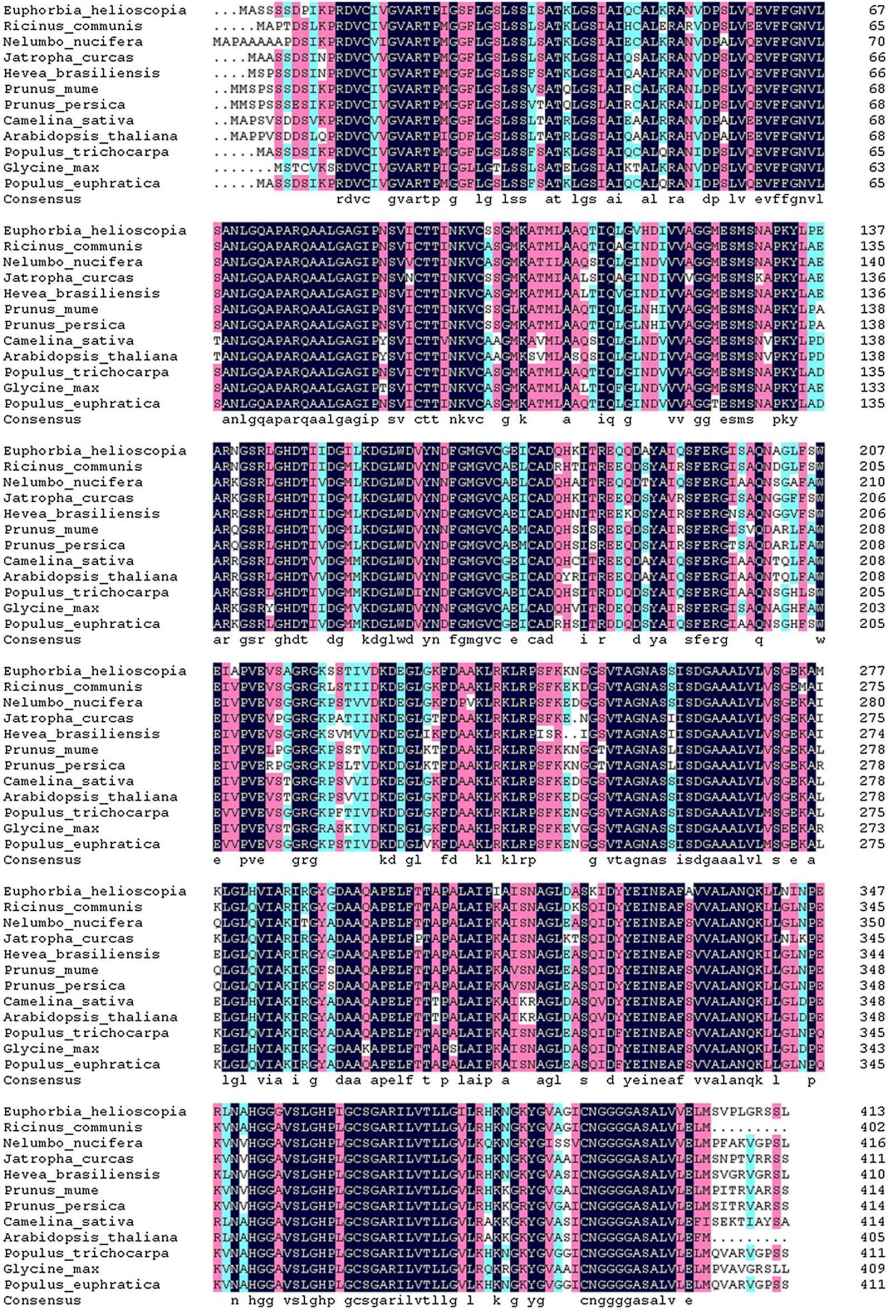
Multiple sequence alignment of deduced *EhAACT* with those of other species. Blue shading means 100% sequence identity, pink shading means more than 75% sequence identity, light blue means more than 50% sequence identity; underline means specific domain of thiolase

Homologous analysis on a database searching with Blast at http://blast.ncbi.nlm.nih.gov/Blast.cgi and the DNAMAN Tool showed that the *EhAACT* shared above 85% identities with *Ricinus communis*, *Jatropha curcas*, *Populus euphratica* and *Hevea brasiliensis* respectively. The *AACT* sequences of other 11 species of highly homology from GenBank were taken for multiple sequence alignment analysis (Fig. 2). The result indicated that C-terminal was more conservative than N-terminal.

**Fig. 3:**
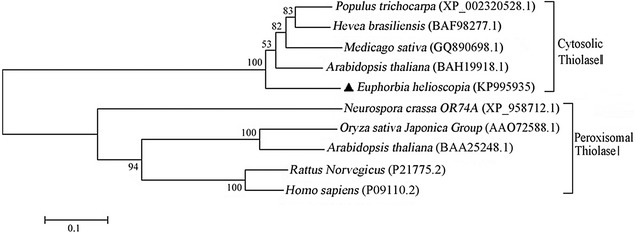
The phylogenetic tree of acetoacetyl-CoA thiolase (AACT) was constructed with the neighbor-joining method of the program MGEA 6.0 and reflected functional classification of thiolases by comparing with other species. Bootstrap value was calculated with 1000 permutations to identify the homogeneity of the analysis. *Euphorbia helioscopia* AACT exhibited highly functional similar with *Populus trichocarpa*, *Hevea brasiliensis*, *Medicago sativa* and *Arabidopsis Thaliana*. And it displayed a more distant relationship with protein of AACT from other species, including *Neurospora crassa* OR74A, *Oryza sativa Japonica* Group, *Arabidopsis thaliana*, *Rattus Norvegicus* and *Homo sapiens*

In order to further determine the EhAACT function, the amino acid sequence of EhAACT was compared with amino acid sequences of other species by phylogenetic tree (Fig. 3). The result showed that AACT was functionally divided into two main branches including MVA cytosolic thiolases and peroxisomal thiolases. Cytosolic thiolases known as thiolase II can catalyze two acetyl-CoA molecules to form an acetoacetyl-CoA in the first step of MVA pathway (Dyer et al. 2009). Whereas, peroxisomal thiolases called thiolases I plays a significant role in β-oxidation process of fatty acid (Hooks 2002). EhAACT were clustered into the same branch with thiolase II of *Populus trichocarpa*, *Hevea brasiliensis*, *Medicago sativa* and *Arabidopsis Thaliana* in evolutionary tree, which exhibited highly functional similarity. And it displayed functional difference with thiolases I from *Neurospora crassa* OR74A, *Oryza sativa Japonica* Group, *Arabidopsis thaliana*, *Rattus Norvegicus* and *Homo sapiens*. The *Arabidopsis thaliana* AACT (BAH19918.1-AT5G48230-acat2) had been reported to locate in the cytosol by subcellular localization of fusion proteins of AACT and GFP, and play an important role in process of terpenoid biosynthesis as a thiolase II (Ahumada et al. 2008). In addition, the protein of AACT1 of *Medicago sativa* (MsAACT1) known as a thiolase II was also found to locate in cytosol and regulated the MVA pathway during abiotic stress adaptation (Soto et al. 2011). In *Hevea brasiliensis*, the gene of acetyl-CoA acetyltransferase has been identified and it was highly expressed in latex, its protein was also functionally classified as a thiolase II and was thought to be a major enzyme in the process of terpenoid biosynthesis (Sando et al. 2008). Therefore, from stated above, it can be verified that the EhAACT belongs to thiolase II on the function and catalyzes terpenoid biosynthesis in MVA pathway.

### Organ distribution of *EhAACT*

**Fig. 4:**
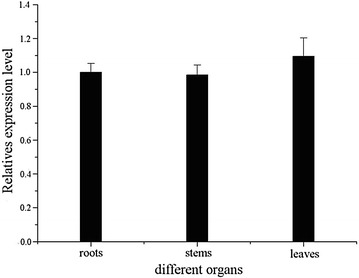
The relative expression levels of *EhAACT* gene in the roots, stems and leaves of *Euphorbia helioscopia*. There was similar expression level among roots, leaves and stems of *EhAACT* and no significant difference (*P* < 0.05) among them

The mRNA transcripts of *EhAACT* were detected by real-time RT-PCR (Fig. 4). The *EhAACT* expression level was similar in roots and stems, and little higher in leaves, and there was no significant difference (*P* < 0.05). The expression of *AACT* gene which was cloned from *Houttuynia cordata* was highest in stem, followed by the underground stem, the expression in flowers and leaves was relatively low (Yao et al. 2015). The transcription level of *SmAACT* was relatively higher in roots than that in stems and leaves in *Salvia miltiorrhiza* (Cui et al. 2010). Therefore, mRNAs for individual pathway enzymes or isozymes can accumulate to significantly different levels in the different organs or developmental stages (Vranová et al. 2013).

### Prokaryotic expression and antibody preparation

**Fig. 5:**
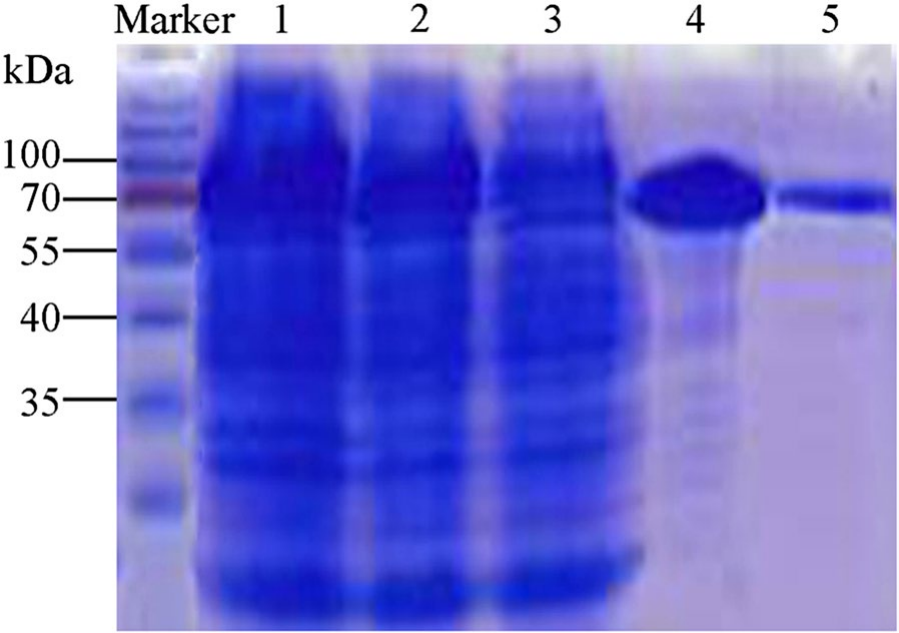
SDS-PAGE analysis of prokaryotic expression products of EhAACT. Marker: Protein marker; 1: All the bacterial solution of prokaryotic expression; 2: Bacterial solution produced by IPTG induction; 3: The supernatant of breaking bacterial cells; 4: Fusion protein produced by Ni-chelating affinity chromatography; 5: Protein solution diluted tenfolds

It was successful for the prokaryotic expression by using a complete open reading frame of *EhAACT*. All the bacterial solution of prokaryotic expression, the bacterial fluid after induced by IPTG, the supernatant after flow, and the protein solution after affinity chromatography of nickel column was detected by the method of SDS-PAGE (Fig. 5). The molecular weight of Ni column affinity sample containing GST-His-EhAACT recombinant protein product of *E. helioscopia* is about 86 kDa. When vector fragment of 43 kDa is removed, the remaining is about 43 kDa which is the molecule weight of recombinant EhAACT protein and extremely close with the EhAACT molecule weight predicted (42.61 kDa).

**Fig. 6:**
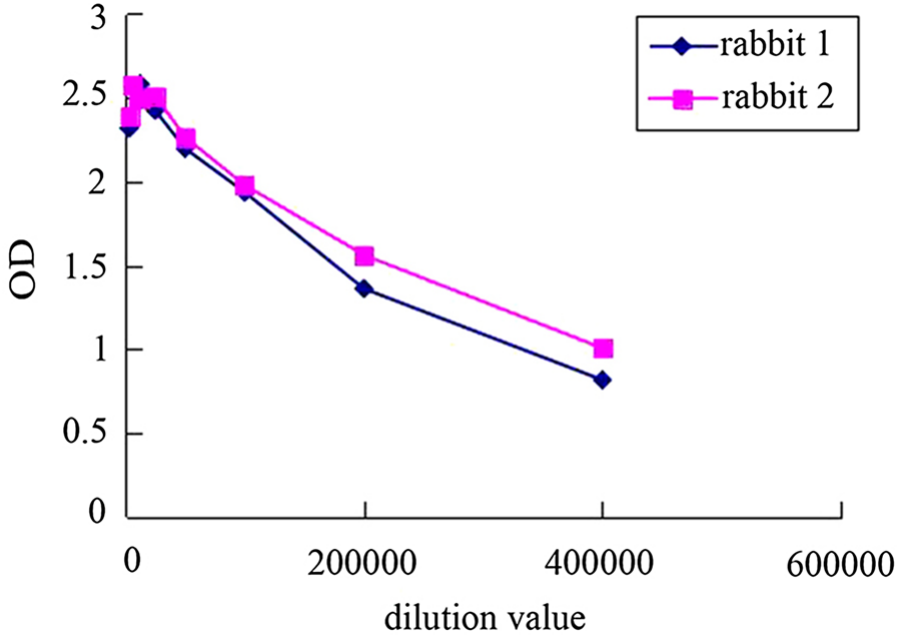
The curve of antiserum titer detected by indirect ELISA

The purified AACT protein was used as antigen to immunize rabbits. After seven times of immunization, the serum was taken to obtain antibodies. The concentration of the protein antigen in the experiment was about 1 μg/mL. The titer of antibody indicates the effective concentration of antibody, which was an important index of antibody quality detection. The antibody titer was detected by indirect ELISA, and the results were shown in Fig. 6. The titer of antibody is about 400 K, which indicates that our EhAACT antibody is qualified to use in the following immunolocalization experiment.

### Immunoblotting analysis of EhAACT antibodies

**Fig. 7:**
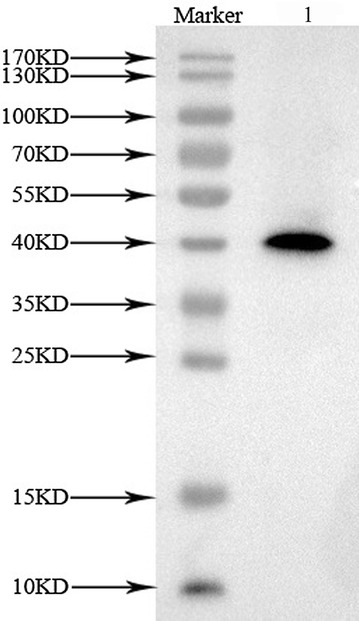
Immunoblot analysis with EhAACT antibody. Lane 1: marker; Lane 2: the corresponding protein gel blot probed with affinity-purified anti-EhAACT antibody

In order to verify if EhAACT functions as endogenous AACT in *E. helioscopia* laticifers, western blotting was proceeded. Immunoblotting analysis found out an obvious band appearing at little above 40 kDa which corresponds to the relative molecular mass 43 kDa of AACT (Fig. 7), and showed that the EhAACT antibodies specifically recognized the endogenous proteins. In addition, this result also proved that the anti-EhAACT antibody is highly specific.

### Subcellular localization of AACT in laticifers

**Fig. 8:**
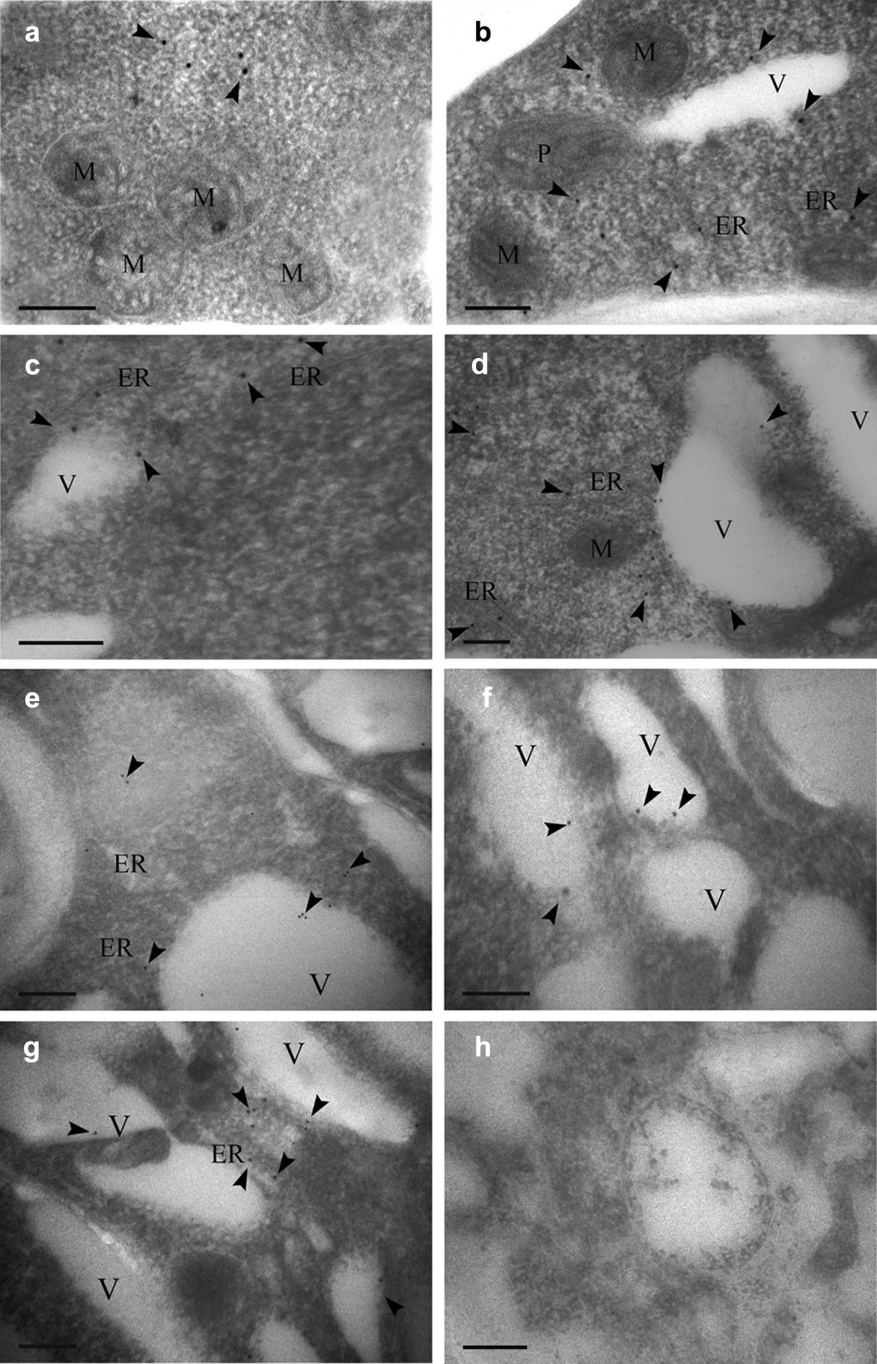
Immunolocalization of the AACT in *Euphorbia helioscopia* laticifers. **a** AACT signals appeared to locate in the cytosol randomly (arrows). **b** AACT signals appeared to locate mainly in endoplasmic reticulum and cytosol near small vacuoles from endoplasmic reticulum (arrows). **c** AACT signals appeared to locate in the endoplasmic reticulum, cytosol near small vacuoles from endoplasmic reticulum (arrows). **d** Golden particles conjugated AACT antibodies were found to locate in the endoplasmic reticulum, cytosol near small vacuoles from endoplasmic reticulum (arrows). **e** AACT signals locate in small vacuoles from endoplasmic reticulum and cytosol (arrows). **f** AACT signals locate in small vacuoles from endoplasmic reticulum (arrows). **g** Golden particles conjugated AACT antibodies were found to locate in the endoplasmic reticulum, small vacuoles from endoplasmic reticulum and cytosol between small vacuoles (arrows). **h** Negative controls showed no gold labeling in the laticifer cells. Bars = 200 nm. ER, Endoplasmic reticulum; M, Mitochondrion; P, Plastid; V, Vacuole

Laticifers were polygonal, and their cell wall was thickened obviously, these characteristics make them easier to be identified from the other cells around. Our present studies showed that in the early development of laticifers, cytoplasm is relatively uniform, there are a large number of mitochondria with distinct cristae (Fig. 8a, b), and rich endoplasmic reticulums dispersed in the cytoplasm (Fig. 8b–d). Colloidal gold particles were observed to locate in the cytosol (Fig. 8a, d), endoplasmic reticulum cisterna (Fig. 8b–d), the cytosol around endoplasmic reticulum, and small vacuoles from endoplasmic reticulum (Fig. 8c, d). With the development of laticifers, it could be seen that endoplasmic reticulum cisternae swelled to form lots of vacuoles, and these vacuoles were always separated by cytosol, colloidal gold particles were distributed around endoplasmic reticulum and mainly in these vacuoles (Fig. 8e, f). Some colloidal gold particles were also observed to locate in cytosol and endoplasmic reticulum between vacuoles (Fig. 8g). In the negative control which was not treated with the primary antibody incubation, no colloidal gold particles were found (Fig. 8h), which indicated that the positive reaction in our experiment is reliable. In the past decades of years, many of the mammalian MVA pathway enzymes were mostly reported to locate in peroxisomes (Kovacs et al. 2002, 2007). AACT was also identified as a peroxisomal protein in mammals (Thompson and Krisans 1990; Olivier et al. 2000). However, Hogenboom et al. (2004) found a cytosolic localization of endogenous human MK using immunofluorescence microscopy and immunocytochemistry, the postulated role of peroxisomes in isoprenoid biosynthesis has been challenged (Hogenboom et al. 2004). In plants, Sapir-Mir et al. (2008) pointed out a new model for compartmentalization of the plant MVA pathway in the three cellular compartments: cytosol, ER, and peroxisome. Endogenous *Arabidopsis* HMGR was observed to locate in steady state within ER and predominantly within spherical, vesicular structures that range from 0.2- to 0.6-mm diameter, and locate in the cytoplasm and within the central vacuole in differentiated cotyledon cells (Leivar et al. 2005). Expression of green fluorescent protein (GFP)-tagged versions of squalene synthase (SQS) in onion epidermal cells demonstrated that SQS1 is targeted to the endoplasmic reticulum (ER) membrane and that this location is exclusively dependent on the presence of the SQS1 C-terminal hydrophobic trans-membrane domain (Busquets et al. 2008). AACT was recently identified as a peroxisomal protein by a proteomic approach of *Arabidopsis* (Reumann et al. 2007). In the model of Sapir-Mir et al., AACT were also considered as peroxisomal localization (Sapir-Mir et al. 2008). While, in *Arabidopsis thaliana*, a member of the AACT family called acat2 has been characterized with cytosolic location (Carrie et al. 2007; Ahumada et al. 2008). Ahumada et al. (2008) reported the characterization of two genes from *Arabidopsis thaliana*, ACT1 and ACT2, which encode two closely related AACT isoforms (Ahumada et al. 2008). Transient expression of constructs encoding AACT1 and AACT2 fused to GFP revealed that the two proteins show a different subcellular localization. AACT1 is found in peroxisomes, AACT2 locate in the cytosol and the nucleus. These obtained results are in agreement with the involvement of AACT2 in catalyzing the first step of the MVA pathway. We found EhAACT involving in the MVA pathway was mainly distributed in ER and vacuoles dilated from ER in *E. helioscopia* laticifers by immunogold electron microscopy, which is consistent with reports about HMGR in *Arabidopsis* (Leivar et al. 2005) and SQS1 in onion epidermal cells (Busquets et al. 2008). In addition, we also found diphosphomevalonate decarboxylase (MDC), the last rate-limiting enzyme of generating the IPP of terpenoid in MVA pathway, to locate mainly in the cisternae of endoplasmic reticulum and small vacuoles from endoplasmic reticulum in *E. helioscopia laticifers* (Chai et al. 2017).

Furthermore, Groeneveld purified latex particles from laticifers of a number of *Euphorbias* species by using gel filtration chromatography and showed that the latex particles were composed of triterpenoids (Groeneveld 1976). Besides, latex particles were also found to be triterpenes in *E. lathyris* latex (Skrukrud 1987). Moreover, some reports pointed out that the latex particles were synthesized in vacuoles originated from ER (Fineran 1983; Skrukrud 1987; Cai et al. 2009). Therefore, it can be say that the latex particles are the accumulation site of terpenoid end products in the laticifers, and the mevalonate to terpenoid converting activity is associated with the structure which was identified as a vacuole originated from ER. Based on stated above, we further speculated that as an initial enzyme in MVA pathway for terpenoid backbone biosynthesis, the EhAACT located in cytosol could be transported to small vacuoles originated from ER to synthesize terpenoid backbones. Lastly, the terpenoid was further synthesized into latex particles in these small vacuoles in *E. helioscopia* laticifers.

## Abbreviations

IPP: isoprene phosphate
DMAPP: dimethylallyl pyrophosphate
MVA: mevalonate
MEP: 2-C-methyl-d-erythritol-4-phosphate
AACT: acetyl-CoA acetyltransferase
ER: endoplasmic reticulum
RT-PCR: reverse transcription-polymerase chain reaction
MK: mevalonate kinase
HMGR: 3-hydroxy-3-methyl glutaryl CoA reductase
SQS: squalene synthase
GFP: green fluorescent protein
MDC: diphosphomevalonate decarboxylase
